# The Use of Cannabinoids for Insomnia in Daily Life: Naturalistic Study

**DOI:** 10.2196/25730

**Published:** 2021-10-27

**Authors:** Nirushi Kuhathasan, Luciano Minuzzi, James MacKillop, Benicio N Frey

**Affiliations:** 1 Mood Disorders Program and Women’s Health Concerns Clinic St. Joseph’s Healthcare Hamilton Hamilton, ON Canada; 2 Michael G DeGroote Centre for Medicinal Cannabis Research McMaster University Hamilton, ON Canada; 3 Department of Psychiatry and Behavioural Neurosciences McMaster University Hamilton, ON Canada; 4 Peter Boris Centre for Addictions Research McMaster University/St. Joseph’s Healthcare Hamilton Hamilton, ON Canada

**Keywords:** medicinal cannabis, insomnia, symptom management, linear mixed-effects

## Abstract

**Background:**

Insomnia is a prevalent condition that presents itself at both the symptom and diagnostic levels. Although insomnia is one of the main reasons individuals seek medicinal cannabis, little is known about the profile of cannabinoid use or the perceived benefit of the use of cannabinoids in daily life.

**Objective:**

We conducted a retrospective study of medicinal cannabis users to investigate the use profile and perceived efficacy of cannabinoids for the management of insomnia.

**Methods:**

Data were collected using the Strainprint app, which allows medicinal cannabis users to log conditions and symptoms, track cannabis use, and monitor symptom severity pre- and postcannabis use. Our analyses examined 991 medicinal cannabis users with insomnia across 24,189 tracked cannabis use sessions. Sessions were analyzed, and both descriptive statistics and linear mixed-effects modeling were completed to examine use patterns and perceived efficacy.

**Results:**

Overall, cannabinoids were perceived to be efficacious across all genders and ages, and no significant differences were found among product forms, ingestion methods, or gender groups. Although all strain categories were perceived as efficacious, predominant *indica* strains were found to reduce insomnia symptomology more than cannabidiol (CBD) strains (estimated mean difference 0.59, SE 0.11; 95% CI 0.36-0.81; adjusted *P*<.001) and predominant *sativa* strains (estimated mean difference 0.74, SE 0.16; 95% CI 0.43-1.06; adjusted *P*<.001). Indica hybrid strains also presented a greater reduction in insomnia symptomology than CBD strains (mean difference 0.52, SE 0.12; 95% CI 0.29-0.74; adjusted *P*<.001) and predominant sativa strains (mean difference 0.67, SE 0.16; 95% CI 0.34-1.00; adjusted *P*=.002).

**Conclusions:**

Medicinal cannabis users perceive a significant improvement in insomnia with cannabinoid use, and this study suggests a possible advantage with the use of predominant indica strains compared with predominant sativa strains and exclusively CBD in this population. This study emphasizes the need for randomized placebo-controlled trials assessing the efficacy and safety profile of cannabinoids for the treatment of insomnia.

## Introduction

### Background

With the growing interest in the therapeutic and medicinal uses of cannabis, there is an increased need to better understand the harms and benefits of acute and long-term therapeutic use of cannabinoids. Among individuals who use medicinal cannabis in Canada, 42% report using cannabis 2-3 times a day, with 40% of users reporting their consumption to be >14 grams per week [1]. In fact, rates of medicinal cannabis authorization in Canada rose from 8000 in 2014 to 340,000 in 2018 [2]. Similarly, with nationwide cannabis legalization in October 2018, general cannabis use rates in Canada increased from 14% to 18% between 2018 and 2019 [3].

Despite the paucity of randomized placebo-controlled trials, both recreational and medicinal cannabis users report perceptions of a broad spectrum of benefits from cannabis. Among these benefits of the use of cannabis is aiding sleep [4]. In fact, in addition to pain and anxiety, insomnia has commonly been reported to be among the top reasons individuals seek medicinal cannabis [2]. This association is very relevant considering the high rates of insomnia in the general population. It is estimated that approximately 10% of adults experience chronic insomnia [5], and nearly one-third of all adults suffer from occasional or intermittent insomnia symptoms annually [5]. Longitudinal studies have found that nearly 70% of individuals reporting insomnia symptoms at baseline continue to report symptoms a year later [6], and 50% continue to report having symptoms 3 years later. Insomnia is also one of the most common complaints in primary care, often presenting itself at both symptom and diagnostic levels [6]. Characterized by difficulty in falling asleep, staying asleep, or having a nonrestorative sleep, insomnia negatively affects functioning, quality of life, and mental health [7]. In addition, insomnia often co-occurs with common medical and psychiatric conditions [6]. Individuals experiencing these comorbidities report greater impairments in psychosocial and cognitive functioning compared with individuals without sleep disturbances [6-10].

### Cannabinoids for Insomnia

Recent reviews have concluded that the current evidence of the benefits of using cannabinoids for insomnia symptoms are largely driven by clinical trials that used cannabinoids for the treatment of other conditions, such as pain or multiple sclerosis [11-13]. Similarly, although some previous studies have examined recreational and medicinal cannabis use in naturalistic samples, very few have focused on insomnia as a primary outcome [14,15]. In one study, 95 medicinal cannabis users were surveyed on the effects of cannabis products used for various conditions and symptoms [14]. The results indicated a statistically significant preference toward *Cannabis indica* products to help with sedation and sleep [14]. In addition, the same study reported that users also preferred these products for insomnia, encouraging further research focusing on the condition [14]. In another study, a mobile app collecting data on medicinal cannabis in naturalistic conditions was used to measure the self-reported effectiveness and side effects of cannabis [15]. The study examined 2332 users across 10,535 tracked cannabis sessions [15]. The results indicated significant reductions in symptom severity across all reported symptoms, with significantly more relief in anxiety- and depression-related symptoms than pain symptoms [15]. Notably, in this particular study, insomnia was examined as a symptom of anxiety and presented the largest symptom relief score across all examined symptoms following cannabis consumption [15].

Most relevantly, a recent naturalistic study that examined cannabis use for insomnia in a sample of 409 participants across 1056 sessions reported significant reductions in symptom severity; however, these findings were limited to raw, natural medical cannabis flowers and lacked information on the perceived efficacy of various cannabis product forms. Furthermore, this study was limited by a lack of information on patient demographics, as the information collected from users did not include key demographic data, such as age and gender [16]. Since the legalization of cannabis in Canada in 2018, research regulations for the drug remain quite stringent [1]. Similarly, because of its status as a schedule 1 drug in the United States, it is underinvestigated for therapeutic purposes [17-20]. Therefore, not only is there a major gap in studies assessing insomnia as the primary outcome, but also a lack of scientific literature on the use of cannabis products that are currently being consumed by the general public [11-13]. To help address these gaps, we conducted a retrospective study to investigate the perceived effectiveness of the use of cannabinoids in treating insomnia symptoms in a large, naturalistic sample of Canadians. We also describe the key demographic characteristics of these individuals, such as age and gender distribution, types of cannabinoid use, and methods of ingestion.

## Methods

### Overview of Strainprint App

We conducted a retrospective study examining cannabis use for the management of insomnia symptoms using anonymous archival data obtained from the medicinal cannabis–tracking app Strainprint (Strainprint Technologies Ltd). Strainprint is a Canadian app with a large database of medical and recreational cannabis users with >90 million data points and 2 million reported patient outcomes. The app allows users to track and monitor changes in their symptoms as a function of different doses, strains, and forms of cannabis. It engages users through a loyalty rewards system where users earn points for tracking sessions of cannabis use. Through Strainprint, users are able to record medical conditions, symptoms being treated, methods of ingestion, doses, emotive effects, pre- and postmedication ratings, and cannabis product constituents by batch for each tracked session. Tracked information can also be shared with health care providers. On initial use of the app, individuals are prompted to enter basic demographic information, such as year and month of birth, gender, and the conditions and symptoms that they wish to treat. When individuals are ready to track their medication session, they open the app before using cannabis and select the relevant symptoms they wish to treat from a dropdown list of their previously chosen symptoms. Users are then taken through a set of steps where they are first prompted to rate the severity of their symptoms on a 11-point numeric rating scale (0=least severe and 10=very severe) before medication. Next, individuals select the cannabis they are using by product name and batch. Strainprint prepopulates the app with lab-verified cannabis constituents by batch for all medical cannabis products sold by licensed producers in Canada. Data on cannabis content are pulled directly from cannabis distributors. Users then select the product form (flower, oil, capsule, edible, vape pen, or concentrate), route of administration (vape, oil, smoke, edible, pill, tincture, spray, concentrate, dab bubble, dab portable, oral, topical, or transdermal), and dose (drops, mg, ml, or puffs) for that specific session. After an onset period defined by the chosen route of administration (eg, 20 minutes for smoke and 60 minutes for pill or edible), users are prompted with a push notification (8 hours later for sleep) to complete their session by rating their symptom severity postmedication on the same 11-point numeric scale.

Strainprint also provides individuals with a complete history of their use, along with product recommendations based on other users’ experiences with the same symptoms. As part of Strainprint’s terms of service, individuals agree to share their anonymous information for research and other purposes. In this study, we examined the data of individuals who used medicinal cannabis to manage the severity of insomnia symptoms for the condition of insomnia. Specific variables for this study were determined before data extraction, and the information was subsequently provided by Strainprint stripped of identifiers.

### Study Sample

Our study included all tracked sessions between February 27, 2017, and February 28, 2020. The final sample consisted of 991 Canadian medicinal cannabis users with insomnia who used the app to monitor changes in insomnia symptoms across 24,189 recorded sessions. The sample comprised 42.6% (422/991) self-identified male participants and 56.1% (556/991) self-identified female participants (13/991, 1.3% of users did not report gender), ranging in age from 18 to 74 years (mean 36.32, SD 11.65). Additional descriptive statistics on the sample are presented in Figure S1 of Multimedia Appendix 1.

### Statistical Analysis

First, we completed a descriptive analysis of the data set by generating information on the specific cannabis use profile for the management of insomnia symptoms. In particular, we examined the frequencies of categorical cannabis use variables such as use time of day, strain categories, product forms, and ingestion methods. These data were further stratified to investigate cannabis use trends by both age and gender. For inferential analyses, our primary analysis focused on the perceived efficacy of cannabis for the management of insomnia symptoms. Efficacy was calculated as the change in insomnia symptomology between pre- and postmedication rates, as reported by users.

Generally, this type of statistical modeling would be completed as a standard regression analysis; however, a standard regression analysis assumes that observations are independent. In this particular data set, users reported multiple observations, and a standard regression analysis would not account for between-person variability in tracked sessions across users. Therefore, we used linear mixed-effects modeling, a type of regression model that estimates random effects (accounting for between-subject variability) in addition to standard fixed effects (accounting for within-subject variability) regardless of differences in the number of reported observations per user. In essence, this mixed-modeling method estimates random intercepts and slopes, which are then used to make more accurate inferences at the fixed-effects level without violating the independence assumption.

Assumptions for each model were checked to ensure the validity of the models used. Residual plots were examined and were determined to not deviate from the assumptions of linearity, normality, and homoscedasticity. The assumption of independence was met by accounting for tracked session nesting within participants using mixed-effects models. For this analysis, linear mixed-effects modeling was used to predict changes in the perceived efficacy of cannabis use with regard to demographic information (ie, age and gender) and cannabis use information (ie, use time of day, product form, and strain category) across tracked sessions. In addition, although several studies have challenged the labeling of strain categories in commercial products, in this naturalistic study, we analyzed this variable as a commercialized label influencing purchasing choices. In all analyses, *P* values were corrected for multiple comparisons using the stringent Bonferroni correction (*P*<.05, Bonferroni corrected).

### Ethical Approval

Ethical approval for this research was granted by the Hamilton Integrated Research Ethics Board (project #7162). The study was designed to be compliant with the Health and Information Protection Act, 2016.

## Results

### Strain Categories for Insomnia

Descriptive statistics examining the percentage of each strain category (ie, predominant sativa, sativa hybrid, predominant indica, indica hybrid, balanced hybrid, or cannabidiol [CBD]) used for the management of insomnia symptoms across 24,189 tracked sessions are presented in Table 1. Overall, predominant indica and indica hybrid strains were the most commonly used strains for insomnia, whereas predominant sativa and sativa hybrid strains were used least for the management of insomnia symptoms. Notably, although CBD is not traditionally considered a strain category, Strainprint recognizes the variations in tetrahydrocannabinol (THC) or CBD content across different strains and presents a CBD-predominant product category as a strain on the app. Further descriptive statistics of strain categories stratified by age and gender are presented in Figures S2 and S3 in Multimedia Appendix 1.

**Table 1:** Descriptive information on frequency of strain categories used across 24,189 tracked sessions (N=24,189).

Stain category	Sessions, n (%)
Indica	9263 (38.29)
Indica hybrid	6468 (26.74)
CBD^a^	3327 (13.75)
Balanced hybrid	3068 (12.68)
Sativa	1098 (4.54)
Sativa hybrid	605 (2.5)

^a^CBD: cannabidiol.

### Cannabis Product Forms and Ingestion Methods for Insomnia

Descriptive statistics examining the frequencies of cannabis product forms (ie, flower, oil, capsule, edible, vape pen, or concentrate) used for the management of insomnia symptoms across 24,189 tracked sessions are presented in Table 2. Because of the relatively small number of data points, products in the form of vape pens and concentrates were combined to form an *other* group. Across all age groups and genders, cannabis was most often used in the form of flowers, followed by oil products, for the management of insomnia symptoms. Table 3 presents descriptive statistics examining the frequencies of cannabis ingestion methods (ie, vape, oil, smoke, edible, pill, tincture, spray, concentrate dab bubbler, dab portable, oral, topical, or transdermal) across all tracked sessions. Again, because of the relatively small number of data points, the categories of concentrate, dab bubbler, dab portable, oral, topical, and transdermal were combined to form a single category. Vaping was the most popular ingestion method across all age groups and genders. All reported results were stratified by age and gender.

**Table 2:** Frequency and percentage of cannabis product forms used across 24,189 sessions between genders and age groups (N=24,189).

Product form	Sessions, n (%)										
	By gender				By age (years)						Overall
	Female	Male	Unknown	18-24		25-34	35-44	45-54	>55		
Flower	6767 (43.82)	8517 (55.2)	160 (1.04)	1712 (11.09)		3954 (25.6)	5622 (36.4)	1934 (12.5)	1997 (12.9)	15,444 (100)	
Oil	5407 (70.2)	2222 (28.9)	70 (0.9)	336 (4.4)		1332 (17.3)	2401 (31.2)	1609 (20.9)	1887 (24.5)	7699 (100)	
Capsule	793 (94.5)	44 (5.2)	2 (0.2)	1 (0.1)		341 (40.6)	179 (21.3)	47 (5.6)	269 (32.1)	839 (100)	
Edible	121 (96)	5 (4)	0 (0)	15 (11.9)		21 (16.7)	6 (4.8)	0 (0)	73 (57.9)	126 (100)	
Other	36 (44.4)	45 (55.6)	0 (0)	2 (2.5)		6 (7.4)	38 (46.9)	18 (22.2)	17 (21)	81 (100)	

**Table 3:** Frequency and percentage of cannabis ingestion methods across 24,189 sessions between genders and age groups (N=24,189).

Ingestion method	Sessions, n (%)										
	By gender				By age (years)						Overall
	Female	Male	Unknown	18-24		25-34	35-44	45-54	>55		
Vape	3985 (46.9)	4419 (52)	91 (1.1)	719 (8.5)		1773 (20.9)	3180 (37.4)	1315 (15.5)	1389 (16.4)	8495 (100)	
Oil	5277 (71.4)	2044 (27.7)	68 (0.9)	321 (4.3)		1282 (17.4)	2208 (29.9)	1570 (21.2)	1840 (24.9)	7389 (100)	
Smoke	2379 (38.3)	3774 (60.7)	62 (1)	947 (15.2)		2062 (33.2)	2186 (35.2)	438 (7)	521 (8.4)	6215 (100)	
Edible	546 (58.6)	383 (41.1)	3 (0.3)	37 (4)		165 (17.7)	425 (45.6)	135 (14.5)	152 (16.3)	932 (100)	
Pill	635 (93.5)	44 (6.5)	0 (0)	2 (0.3)		255 (37.6)	96 (14.1)	46 (6.8)	278 (40.9)	679 (100)	
Tincture	121 (77.1)	32 (20.4)	4 (2.5)	12 (7.6)		37 (23.6)	41 (26.1)	54 (34.4)	12 (7.6)	157 (100)	
Spray	108 (85.7)	15 (11.9)	3 (2.3)	0 (0)		16 (12.7)	51 (40.5)	25 (19.8)	32 (25.4)	126 (100)	
Other	73 (37.2)	122 (62.2)	1 (0.5)	28 (14.3)		64 (32.7)	59 (30.1)	25 (12.8)	19 (9.7)	196 (100)	

### Symptom Severity Ratings

**Figure 1:**
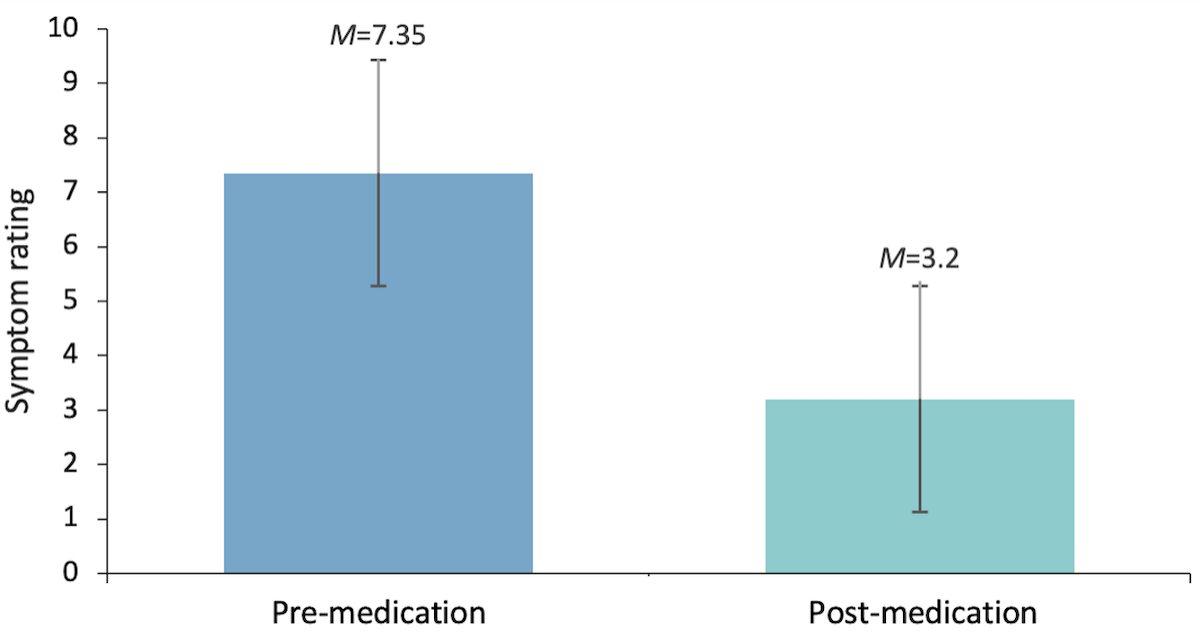
Mean symptom severity ratings pre- (mean 7.35, SD 1.88) and post-(mean 3.20, SD 2.37) cannabis use.

Mean symptom severity ratings were examined before and after cannabis use across tracked sessions (N=991 users across 24,819 sessions; Figure 1). Before cannabis use, the mean symptom severity rating across sessions was 7.35 (SD 1.88), whereas the mean symptom severity rating after use was 3.20 (SD 2.37).

### Linear Mixed-Effects Model Predictions of Perceived Efficacy

We first examined the perceived efficacy of cannabinoid use for insomnia as a function of gender and found it to be significant across both genders (Table 4). The perceived efficacy of cannabinoid use for insomnia was also significant across all age groups (Table 5). Comparisons between gender and age can be found in Multimedia Appendix 1 (Tables S1 and S2).

**Table 4:** Efficacy by gender. The efficacy was tested using linear mixed modeling (β coefficient was not standardized).

Gender	Estimate (SE)	*t* test^a^ (*df*)	*P* value
Female	3.4289 (0.1696)	26.814 (852.4)	<.001
Male	3.5282 (0.1288)	27.399 (868.3)	<.001

^a^Tests were two-tailed.

**Table 5:** Efficacy by age groups. The efficacy was tested using linear mixed modeling (β coefficient was not standardized).

Age (years)	Estimate (SE)	*t* test^a^ (*df*)	*P* value
18-24	3.43291 (0.16118)	21.298 (2073.5)	<.001
25-34	3.35234 (0.11098)	30.206 (1656.2)	<.001
35-44	3.72108 (0.10826)	34.370 (1604.7)	<.001
45-54	3.12995 (0.13658)	22.916 (1894.7)	<.001
>55	3.76299 (0.15390)	24.251 (2242.4)	<.001

^a^Tests were two-tailed.

Next, we examined whether the time of cannabis use predicted perceived efficacy and found that efficacy was significant regardless of the time of day (Table 6). Because of the nature of the data, information on shift work was not available; therefore, we did not compare efficacy across different use times during the day. More detailed frequency and percentage information of sessions for each use time of the day can be found in Table S3 of Multimedia Appendix 1.

**Table 6:** Efficacy by use time of day. The efficacy was tested using linear mixed modeling (β coefficient was not standardized).

Time of day	Estimate (SE)	*t* test^a^ (*df*)	*P* value
Morning	3.668345 (0.102629)	35.744 (488.1)	<.001
Afternoon	2.754512 (0.225454)	12.218 (120.1)	<.001
Evening	3.369924 (0.119686)	28.156 (391.6)	<.001
Overnight	3.449696 (0.089454)	38.564 (739.5)	<.001

^a^Tests were two-tailed.

We also examined perceived efficacy as a function of cannabis product forms and found that all product forms were perceived as efficacious (Table 7). Notably, for some product forms (ie, vape pen and concentrate), there were too few observations to warrant inclusion in primary analyses, even when combined to form a single category. Therefore, of all available product forms, only those making up at least 0.005% of the data set were included in the analyses. There were no significant differences in efficacy among product forms (all *P*>.05; Table S4, Multimedia Appendix 1).

**Table 7:** Efficacy by product form. The efficacy was tested using linear mixed modeling (β coefficient was not standardized).

Product form	Estimate (SE)	*t* test^a^ (*df*)	*P* value
Capsules	3.79417 (0.34792)	10.9054 (25.7)	<.001
Edible	4.15951 (0.56358)	7.3806 (7.9)	<.001
Flower	3.43969 (0.08728)	39.4100 (737.0)	<.001
Oil	3.47823 (0.11693)	29.7470 (374.8)	<.001

^a^Tests were two-tailed.

Finally, we examined perceived efficacy as a function of strain category and found that cannabis was efficacious regardless of the specific strain being used (Table 8). Interestingly, predominant indica strains were found to be more efficacious than CBD (estimated mean difference 0.59, SE 0.11; 95% CI 0.36-0.81; adjusted *P*<.001) and predominant sativa strains (estimated mean difference 0.74, SE 0.16; 95% CI 0.43-1.06; adjusted *P*<.001). Indica hybrid strains were also found to be more efficacious than CBD (estimated mean difference 0.52, SE 0.12; 95% CI 0.29-0.74; adjusted *P*<.001) and predominant sativa strains (estimated mean difference 0.67, SE 0.16; 95% CI 0.34-1.00; adjusted *P*=.002). Balanced hybrid strains were also found to be more efficacious than CBD (estimated mean difference 0.39, SE 0.13; 95% CI 0.14-0.64; adjusted *P*=.03) and sativa strains (estimated mean difference 0.54, SE 0.17; 95% CI 0.20-0.88; adjusted *P*=.03; Table 9).

**Table 8:** Efficacy by strain categories. The efficacy was tested using linear mixed modeling (β coefficient was not standardized).

Strain categories	Estimate (SE)	*t* test^b^ (*df*)	*P* value
Balanced hybrid	3.461359 (0.112701)	30.713 (300.8)	<.001
CBD^a^	3.074027 (0.114947)	26.743 (367.7)	<.001
Indica	3.661426 (0.094507)	38.742 (673.9)	<.001
Indica hybrid	3.589259 (0.097939)	36.648 (469.1)	<.001
Sativa	2.916945 (0.163117)	17.883 (86.4)	<.001
Sativa hybrid	3.470149 (0.171074)	20.285 (92.0)	<.001

^a^CBD: cannabidiol.

^b^Tests were two-tailed.

**Table 9:** Efficacy comparisons between strain categories. The efficacy was tested using linear mixed modeling (β coefficient was not standardized).

Strain categories	Estimate (SE)	*t* test^b^ (*df*)	*P* value
Balanced hybrid versus CBD^a^	0.387332 (0.125752)	3.080 (167.3)	.03
Indica versus balanced hybrid	0.200067 (0.107863)	1.855 (208.6)	.98
Indica hybrid versus balanced hybrid	0.127900 (0.104839)	1.220 (128.9)	.99
Balanced hybrid versus sativa	0.544414 (0.170767)	3.188 (72.0)	.03
Sativa hybrid versus balanced hybrid	0.008790 (0.181109)	0.049 (75.1)	.99
Indica versus CBD	0.587400 (0.114173)	5.145 (216.2)	<.001
Indica hybrid versus CBD	0.515232 (0.115805)	4.450 (197.1)	<.001
CBD versus sativa	0.157082 (0.163729)	0.959 (58.6)	.99
Sativa hybrid versus CBD	0.396122 (0.181141)	2.187 (82.9)	.48
Indica versus indica hybrid	0.072168 (0.083424)	0.865 (183.5)	.99
Indica versus sativa	0.744481 (0.159664)	4.663 (69.6)	<.001
Indica versus sativa hybrid	0.191277 (0.162713)	1.176 (68.4)	.99
Indica hybrid versus sativa	0.672314 (0.164905)	4.077 (73.6)	<.001
Indica hybrid versus sativa hybrid	0.119110 (0.166813)	0.714 (82.5)	.99
Sativa hybrid versus sativa	0.553204 (0.201776)	2.742 (65.5)	.12

^a^CBD: cannabidiol.

^b^Tests were two-tailed.

## Discussion

### Principal Findings

Results from this large naturalistic sample of medicinal cannabis users who tracked their insomnia symptoms before and after cannabis use suggest significant improvements in insomnia symptoms, with no gender differences in perceived efficacy. Notably, this study uses a naturalistic design by analyzing crowdsourced data from a medicinal cannabis–tracking mobile app. With increasing advances in technology, this study presents a unique perspective on a health management self-monitoring tool that examines data on a population scale.

Analyses of product forms and ingestion methods found that cannabis was most often used in the form of flowers or oils and most often ingested via vapes, oils, or smoking. In addition, although all strains were reported to be beneficial for the management of insomnia, predominant indica and indica hybrid strains were found to be more efficacious than CBD and predominant sativa strains. This finding is in contrast with those of a previous study reporting that strains with significantly higher concentrations of CBD were generally preferred by individuals using cannabis to treat symptoms of insomnia [21]. Despite this, our findings are in line with results from previous studies that have reported indica and hybrid strains to be among the most frequently used strains for insomnia [16]. This same study reported that the most used strains were fairly high in THC content and were combined with high to moderate CBD content.

Another study investigating multiple doses of cannabinoids for sleep reported that administration of both 5 mg/5 mg and 15 mg/15 mg of THC/CBD demonstrated a decrease in stage 3 sleep when compared with placebo, with the higher dose also showing increased states of wakefulness [22]. THC administration on its own demonstrated no significant changes to sleep architecture from placebo; however, the same study found that high doses of THC alone or in combination with CBD resulted in increased subjective sleepiness [22]. From this, the researchers concluded that CBD may have dose-dependent effects on alertness and that the activating and sedating properties of CBD and THC, respectively, could work together to induce sleep and counteract daytime sleepiness [22]. Although few clinical trials have objectively analyzed cannabinoids for sleep with sleep outcomes as primary measures, some preliminary trials have shown that administration of THC and THC-derivatives, alone or in combination with CBD, were associated with subjective improvement in sleep outcomes [11-13]. In addition, previous studies examining strain preferences have also reported increased preferences toward indica strains for sleep [14,23,24]. In one study, indica was preferred for sedation and sleep, whereas sativa was preferred to increase energy [14]. Another study investigating qualitative responses reported that patients using medicinal cannabis preferred using indica at night to improve sleep [24]. In essence, to better understand the efficacy of cannabinoids for insomnia, randomized placebo-controlled studies are needed.

The human endocannabinoid (eCB) system has been increasingly implicated in body and brain homeostasis, including sleep. For instance, the eCB system is thought to play an active role in regulatory processes, such as pain perception, memory, and sleep modulation [25,26]. Although the neurobiological basis of cannabis for sleep is still being understood, overlaps between the neuronal circuitry of sleep and wake states and the eCB system suggest that cannabinoids can contribute to sleep-related mechanisms and physiology [27-29]. Therefore, the eCB system has become a growing target in sleep research [25-30]. Despite the perceived benefits of cannabinoids, there remains a lack of placebo-controlled trials that have examined the effects of the drug using validated sleep measures or objective sleep outcomes [11-13]. In addition, the current literature on the existence of potential risks, harms, and side effects associated with cannabinoid treatments remain extremely sparse for sleep disorders; however, there is growing evidence that suggests an increased risk of both acute and chronic cognitive impairments [31,32]. Although these risks are poorly understood, research suggests that the prevalence of these effects is increasing [31]. Future clinical trials should focus on the benefits and potential harms through the use of validated objective and subjective measures. Because of the highly comorbid nature of insomnia and other sleep disorders, additional variables such as medication interactions, potential side effects, and comorbid diagnoses are also worth investigating.

### Limitations

Some limitations should be considered when interpreting the results of this study. First, individual conditions and symptoms were subjectively reported by users on the Strainprint app. Therefore, it is unknown whether subjects will meet the full criteria for insomnia or any other sleep-related disorder. Moreover, individual user data are restricted to the information collected by Strainprint; therefore, additional information that may affect cannabinoid efficacy (eg, medical history, body size, other concurrent medications, or tolerance) could not be assessed. Another limitation of this study is the lack of a placebo control group. Because data were collected from a sample of medicinal cannabis users, it is possible that individual expectations of cannabinoid efficacy may have attributed to positive postmedication ratings. In other words, the large magnitude effect-size observed reflects pharmacological effects and response expectancy (placebo) effects, and the proportionate contribution of each, fundamentally, cannot be ascertained. It is also possible that this study examined the data of individuals who were more likely to find cannabis to be effective, as the Strainprint app is geared toward individuals who wish to improve therapeutic outcomes by tracking their cannabis use. As a result, the sample may disproportionately represent users who benefit from using cannabis. In addition, the Strainprint app primarily collects data on cannabis use and has very limited data on its potential side effects. Therefore, beyond perceived efficacy, it was not possible to ascertain from the available data whether users experienced any negative side effects from cannabis use.

This study also examined various strain categories; however, distinctions between these strains remain the subject of much debate [33-35]. Cannabis has historically been classified into two separate species (*C.* *sativa* and *C. indica*) with distinct biological effects. However, years of breeding and hybridization have rendered potential distinctions often meaningless [34-37]. As recreational cannabis use has become increasingly popular, commercialization of the plant has led to the emergence of products marketed as derivatives or hybrids of these species [23,35,36]. Among consumers, the terms *sativa*, *indica*, and *hybrid* are used colloquially and are associated with perceived effects [37]. Sativa has been associated with stimulating effects, indica with sedating effects, and hybrids are perceived to be bred from the former two to fit the more personalized needs of consumers [23,24,35,37]. Interestingly, a recent study collected data characterizing various commercial products classified as *sativa*, *indica*, or *hybrid* and used supervised and unsupervised machine learning algorithms to subjective effect tags [36]. The models indicated a clear division among *sativas* and *indicas*, with *hybrids* in between, suggesting distinct subjective effects among the categories [36].

Despite these perceived effects, strain categories are largely baseless [34-37]. Instead, many researchers hold that the differences in perceived effects between strain categories may be owing to other components of cannabis (ie, terpenes), which are rarely accurately reported to consumers [34,36,38,39]. Interestingly, some studies have found that products labeled as *indica* and *sativa* have similar concentrations of major cannabinoids but distinctly different concentrations of terpenes [23,40,41]. To date, hundreds of different cannabinoids and terpenes have been identified, all with varying pharmacological properties and outcomes [38,42]. These cannabinoids and terpenes are also known to interact synergistically with one another to exert *entourage effects*, which can have enhanced therapeutic benefits for consumers [38,39,42-44]. Given this, it remains unclear whether the perceived therapeutic effects are a result of the individual components of cannabis products or the combined effects of interacting cannabinoids and terpenes. Although strain categories are largely arbitrary, many researchers continue to examine their perceived effects to better understand consumer choices [16,24,44-47]. The nature of our data allowed us to do the same, providing insight into the naturalistic setting of cannabis use. In essence, although the analysis of strain categories in this study provides valuable research on how efficacious various strains are perceived to be, it is worth noting that the perceived efficacy and differences between strains may be driven, at least in part, by self-selection and placebo effects.

Furthermore, previous studies have found inconsistencies between product labels and content, as well as differences in cannabinoid content reporting among labs [48,49]. With recreational cannabis products, the accuracy of product labels relies heavily on growers, suppliers, and dispensaries; however, there are currently no standardized procedures or reliable methods for verifying strains or cannabis content in commercialized products [35]. Despite this, consumers greatly rely on product labels for information on the cannabis content of a product, often using these labels to communicate preferences for desired effects [24,35,36]. One study even reported that demand for *indica* and *sativa* products was similar, with hypothetical purchasing tasks suggesting that consumer decisions were determined by the perceived effects of each strain in the context or setting of the typical activity-based purpose [23]. Unfortunately, because the Strainprint app prepopulates product data from multiple sources, variability across products is an issue, and we were unable to measure the accuracy of cannabinoid content for each product.

For addressing the limitations discussed above, similar future studies should investigate the effects of various terpenes and cannabinoids on perceived efficacy. Previous research has also suggested addressing strain variability by classifying cannabis products according to chemical phenotypes and pharmacological characteristics [35,43,50]. A necessary next step toward accurately classifying cannabis subgroups and creating more precise product labels for consumers is a better understanding of the association between the chemical composition of individual products and the perceived effects experienced by cannabis users. As the colloquial use of strain categories is likely to persist in the commercial marketplace, it is also necessary that future studies attempt to genetically profile samples of commercialized cannabis products, such that genotypes of the same strain are at least comparable. In addition, randomized placebo-controlled trials are necessary to ultimately test the efficacy and safety of cannabis-based treatments for insomnia. Despite these limitations, this study is strengthened by its ecological validity, as data were obtained from a large naturalistic registry of medicinal cannabis users who prospectively tracked changes in their insomnia symptoms before and after cannabis use. The results of this study can help in designing future clinical trials to ultimately test the efficacy and safety profile of different cannabinoids in the management of insomnia.

### Conclusions

The results of this study suggest that individuals using medicinal cannabis to manage insomnia symptoms report significant symptom reduction after use. This general perceived improvement in insomnia symptoms highlights the potential for cannabis to be used as a treatment option for sleep disorders. Future research should investigate the benefits and harms of cannabinoids for insomnia through rigorous randomized placebo-controlled trials.

## Appendix

Multimedia Appendix 1 Supplementary material.

## Abbreviations

CBD: cannabidiol
eCB: endocannabinoid
THC: tetrahydrocannabinol
