# Supplementary material for: The Use of Cannabinoids for Insomnia in Daily Life: Naturalistic Study
Source: J Med Internet Res. 2021 Oct 27;23(10):e25730. doi: 10.2196/25730 (PMC8581757; doi:10.2196/25730)
Supplement: Multimedia Appendix 1 [file jmir_v23i10e25730_app1.docx]

**Supplementary Material**

*FIGURE S1: Descriptive demographic information stratified by age and gender*

*FIGURE S2: Descriptive information on strain categories across 24189 sessions, stratified by gender.*

*FIGURE S3: Descriptive information on strain categories across 24189 sessions, stratified by age.*

**GENDER X EFFICACY COMPARISONS**

|  | Estimate | Std. Error | df | t value | p value |
| --- | --- | --- | --- | --- | --- |
| Male vs. Female | 0.099301 | 0.169602 | 852.4 | 0.5855 | 0.558 |

*TABLE S1: Efficacy comparisons between gender. The efficacy was tested using linear mixed modeling (beta coefficient was not standardized).*

**AGE X EFFICACY COMPARISONS**

|  | Estimate | Std. Error | df | t value | p value |
| --- | --- | --- | --- | --- | --- |
| [18-24] vs. [25-34] | 0.080574 | 0.164535 | 4505.9 | 0.490 | 1.0000 |
| [18-24] vs. [35-44] | -0.288167 | 0.181906 | 3152.4 | -1.584 | 1.0000 |
| [18-24] vs. [45-54] | 0.302963 | 0.205804 | 2628.0 | 1.472 | 1.0000 |
| [18-24] vs. [55+] | -0.330076 | 0.218393 | 2732.1 | -1.511 | 1.0000 |
| [25-34] vs. [35-44] | -0.368740 | 0.117976 | 6311.3 | -3.126 | 0.018** |
| [25-34] vs. [45-54] | 0.222389 | 0.161152 | 3472.9 | 1.380 | 1.0000 |
| [25-34] vs. [55+] | -0.410650 | 0.177985 | 3541/6 | -2.307 | 0.211 |
| [35-44] vs. [45-54] | 0.591129 | 0.137074 | 5784.4 | 4.313 | <.001*** |
| [35-44] vs. [55+] | -0.041910 | 0.159343 | 5626.5 | -0.263 | 1.0000 |
| [45-54] vs. [55+] | -0.633039 | 0.100426 | 21988.3 | -6.304 | <.001*** |

*TABLE S2: Efficacy comparisons between age groups. The efficacy was tested using linear mixed modeling (beta coefficient was not standardized).*

**FREQUENCY OF CANNABIS PRODUCT FORMS**

| Usage Time of Day | Overall |  |
| --- | --- | --- |
| Morning |  | 4306  18% |
| Afternoon |  | 491  2% |
| Evening |  | 5683  23% |
| Overnight |  | 13709  57% |

*TABLE S3: Frequency and percentage of cannabis usage time of day across 24189 sessions.*

**PRODUCT FORM X EFFICACY COMPARISONS**

|  | Estimate | Std. Error | df | t value | p value |
| --- | --- | --- | --- | --- | --- |
| Edible vs. Capsule | 0.365348 | 0.651521 | 13.1 | 0.5608 | 1.0000 |
| Capsule vs. Flower | 0.354477 | 0.341137 | 22,8 | 1.0391 | 1.0000 |
| Capsule vs. Oil | 0.315939 | 0.354712 | 29.0 | 0.8907 | 1.0000 |
| Edible vs. Flower | 0.719824 | 0.558380 | 7.4 | 1.2891 | 1.0000 |
| Edible vs. Oil | 0.681287 | 0.567974 | 8.0 | 1.1995 | 1.0000 |
| Oil vs. Flower | 0.038537 | 0.107118 | 167.4 | 0.3598 | 1.0000 |

*TABLE S4: Efficacy comparisons between product forms. The efficacy was tested using linear mixed modeling (beta coefficient was not standardized).*
